# Age Limit in Bronchiolitis Diagnosis: 6 or 12 Months?

**DOI:** 10.3389/fped.2020.00144

**Published:** 2020-04-20

**Authors:** Raffaella Nenna, Antonella Frassanito, Laura Petrarca, Greta Di Mattia, Fabio Midulla

**Affiliations:** Department of Maternal, Infantile, and Urological Sciences, Sapienza University of Rome, Rome, Italy

**Keywords:** bronchiolitis, infants, respiratory syncyzial virus, wheezing, lower respiratory tract infection

## Abstract

**Aim:** The most frequent cause of lower respiratory tract infection in infants is bronchiolitis. Up to now there is no agreement on the upper limit age of bronchiolitis. Our aim was to identify if there are clinical differences in infants hospitalized for bronchiolitis between 0–6 months and 6–12 months of age. A secondary aim was to establish whether there was differences in terms of recurrent wheezing at 12, 24, and 36 months of follow-up.

**Methods:** We retrospectively analyzed clinical and virological records of 824 infants hospitalized for bronchiolitis during 11 consecutive epidemic seasons. From each infant at admission to the hospital nasopharyngeal washing was collected, clinical severity was assessed and clinical data were extracted from a structured questionnaire. At 12–24–36 months after discharge, parents were interviewed seeking information on recurrent wheezing.

**Results:** A total of 773 infants (Group1) were ≤6 months of age, while 51 were >6 months (Group 2). No differences between family history for atopy and passive smoking exposure were observed between the two groups. Respiratory syncyzial virus was detected more frequently in Group 1 and human bocavirus in Group 2. The clinical severity score (*p* = 0.011) and the use of intravenous fluids (*p* = 0.0001) were higher in Group 1 with respect to Group 2 infants. At 36 months follow-up 163/106 (39.4%) Group 1 and 9/9 Group 2 infants experienced recurrent wheezing (*p* = 0.149).

**Conclusion:** We demonstrated that 0-6 months old infants bronchiolitis differs from > 6 months bronchiolitis.

## Introduction

Bronchiolitis is a common cause of lower respiratory tract infection in infants and the major responsible factor of hospitalization under the age of one year, with the highest incidence occurring between December to March. Respiratory syncytial virus (RSV) is major involved virus, but also other respiratory viruses are able to cause bronchiolitis (1). Some infants that require hospital admission for bronchiolitis will present recurrent wheezing episodes later in life (2). Up to now, there has not been an agreement on bronchiolitis definition concerning the upper age limit, which varies between 12 and 24 months (3, 4). Growing evidences suggest that the upper age limit should be restricted to 6 months (5). We retrospectively analyzed clinical differences between 0–6 months and 6–12 months old infants, among our cohort of 824 infants hospitalized for bronchiolitis at the Pediatric Emergency Department, “Sapienza” University of Rome, during eleven epidemic seasons.

## Materials and Methods

Among our cohort of 824 full-term previously healthy infants hospitalized for bronchiolitis during 11 consecutive epidemic seasons, 773 (93.8%) were 0–6 months of age and 51 (6.2%) were 6–12 months old. Bronchiolitis was defined as the first episode of acute lower airway infection, characterized by a history of upper respiratory tract infection followed by acute onset of respiratory distress with cough, tachypnoea, retraction, and diffuse crackles on auscultation. Exclusion criteria were prematurity and underlying chronic diseases, such as immunodeficiency, hemodynamically significant congenital heart disease, and pulmonary chronic diseases, such as cystic fibrosis and interstitial lung disease.

In line with confidentiality requirements, the database was anonymized and the ethic committees approved the study after infants' parents have released their informed consent. Nasopharyngeal washing (NPW) was collected from each infant within 24 h. Samples were delivered within 1–2 h to the virology laboratory and vortexed with beads to dissolve mucus, if needed. RSV was detected using a panel of reverse transcription PCR or nested PCR assays, as reported (1). At admission to the hospital, clinical severity was assessed using a score ranging from 0 to 8, as described (1). Clinical data were extracted from a structured questionnaire and clinical medical records. At 12-24-36 months after discharge, parents were interviewed using a standardized telephone procedure with a structured questionnaire seeking information on recurrent wheezing (two or more physician verified episodes of wheezing / year for three consecutive years).

A standard statistical package was used for comparisons between groups (SPSS version 21.0, Chicago, IL). χ2 test was used to compare frequency distributions obtained from the two groups. Statistical means, medians, standard deviations and interquartile range were computed as well as Mann-Whitney *U*-test was performed, in order to compare groups. A binary logistic regression analysis was performed to evaluate the odds ratio. A two-tailed *p* < 0.05 was considered significant.

## Results

A total of 773 infants (Group1: median age: 2.0 months, range: 0.2–6.0 months; males: 54.1%) were ≤ 6 months of age, while 51 were >6 months (Group 2: median age: 7.4 months, range: 6.1-12.0 months; males: 47.1%). A family history of asthma (23.5 vs. 17.6%) and atopy (35.3 vs. 35.5%) was similar between the groups. Group 1 infants had slightly less frequently tobacco smoking exposure (*p* = 0.107) than Group 2. The hospitalization for bronchiolitis occurred over the period December-March in the 80.9% of Group 1 and 70.6% of Group 2 infants (*p* = 0.059). A virus was identified from the nasal washing of 49.9% Group 1 and 54.9% of Group 2 infants. Among virus positive infants, RSV was the most frequently detected virus (68.1 vs. 42.9%, *p* = 0.007), followed by human rhinovirus (hRV: 11.7 vs. 14.3%), human bocavirus (hBoV: 1.3 vs. 21.4%, *p* < 0.0001), human metapneumovirus (hMpV: 3.6 vs. 0) and others (3.9 vs. 3.6%); while coinfections were found in 11.4% of Group 1 and 17.9% of Group 2 infants. Among the 575 infants who performed a chest x-ray, a consolidation was found in 55.8% Group 1 and 47.2% Group 2 infants (*p* = 0.202). The clinical severity score (*p* = 0.014), the use of intravenous (IV) fluids (*p* = 0.0001) and the length of hospital stay (*p* = 0.216) were higher in Group 1 with respect to Group 2 infants (Table 1). At 36 months' follow-up 163/106 (39.4%) Group 1 (40.9% of RSV and 47.6% of hRV infants) and 9/9 (50.0%) Group 2 (0 RSV and 100% of hRV) infants experienced recurrent wheezing (*p* = 0.149).

**Table 1:** Clinical characteristics of 824 infants hospitalized for bronchiolitis divided in ≤6 months /> 6 months old infants.

**Items**	**≤ 6 months old infants (*N* = 773)**	**> 6 months old infants (*N* = 51)**	***p***
Male sex	54.1%	47.1%	ns
Passive smoking exposure	45.4%	58.0%	0.107
Virus negative	50.1%	45.1%	ns
Virus positive			
RSV	68.1%	42.9%	0.007
hRV	11.7%	14.3%	ns
hBoV	1.3%	21.4%	<0.0001
hMpV	3.6%	0	ns
Others	3.9%	3.6%	ns
Coinfections	11.4%	17.8%	ns
Clinical severity score median (IQR)	3 (2–5)	3 (2–4)	0.011
IV fluids	55.1%	29.4%	0.001
Days of hospitalization (IQR)	5 (4–6)	4 (4–6)	0.216

*Data were expressed as percentage or median and interquartile range (IQR)*.

*IV, intravenous*.

The bivariate logistic regression designed to investigate the possible confounding factors (sex, clinical severity score, days of hospitalization and exposure to passive smoking, RSV, hBoV, and IV fluid) showed that the 6–12 months group had an odds ratio of hBoV detected at the NPA of 12.84 (95% CI 3.60–45.80) and the need of IV fluid of 0.45 (95% CI 0.20–0.99).

## Discussion

This study analyzed the clinical differences among infants hospitalized with bronchiolitis divided according to the age: ≤ 6 months and > 6 months. Younger infants had more frequently RSV infection and a more severe disease. On the contrary, older infants had slightly more tobacco smoking exposure and slightly higher recurrent wheezing after the hospitalization. The hBoV has been identified almost entirely in infants older than 6 months.

A distinct characteristic of our cohort is the adherence to strict inclusion criteria in bronchiolitis diagnosis, such as age <1 year, infants at the first episode of lower respiratory infection and the presence of crackles. This large series of bronchiolitis infants collected during 11 consecutive epidemic seasons allows an accurate evaluation of the age limit. The definition of bronchiolitis as the first episode of acute viral wheeze occurring in infants less than two years, likely overlaps the early presentation of asthma or bronchial hyper-responsiveness (5). Moreover, bronchiolitis is a frequent and costly disease with mainly supportive treatment. Many studies have been made with the aim of finding other therapies, with controversial results (6). A single definition of bronchiolitis will allow analyzing homogeneously disease's pathogenesis and the possible role of new therapeutic strategies.

Although it has been already demonstrated that clinical presentation of bronchiolitis can vary over different epidemic years (7, 8), the inclusion of full term infants, without any comorbidities over 11 consecutive epidemic seasons in our study should ensure that this variability is overcome.

According to our data, the two age groups differ for the etiology. In fact, RSV remains the most important etiological agent causing bronchiolitis (1), particularly in 0–6 months old infants, while hBoV was almost exclusively detected in older infants. Interestingly, hBoV is an emerging virus frequently identified in wheezing children (9). Moreover, hMpV was detected only in younger children. Some authors indicated that hMPV may cause an illness similar to RSV and follows the same epidemiology (10).

Older infants were slightly more frequently undergone to tobacco smoking exposure. Secondhand tobacco smoke in children causes decreased lung function and increased airway responsiveness and consequently it may predispose infants to more severe infections (11). Passive smoking might be considered as risk factors in this age group. We could conclude that, while bronchiolitis affects independently younger infants, in > 6 months' infants it causes hospitalization particularly when risk factors are present, such as tobacco smoking exposure.

The finding of a more severe disease reflects the different characteristic of younger infants with bronchiolitis, who present with retractions, feeding difficulties, and diffuse crackles and, because of the age are predisposed to a more severe disease. In fact, the leading risk factor for severe bronchiolitis is the young age (12). In addition, 0–6 months old infants had a longer hospital stay. It may be explained because of the more severe illness in this age group. The multivariate analysis showed that the need of IV fluid was higher in the group aged 0–6 months, reflecting a higher severity of presentation in this age group.

According to our findings of a higher occurrence within the epidemic season and of the slightly higher frequency of recurrent wheezing at 36 months' follow-up, infants > 6 months seems represent a different group of patients hospitalized for bronchiolitis, who are more predisposed to childhood asthma. Further studies are needed to clarify the pathogenesis the described difference.

In conclusion, we demonstrated that 0–6 months old infants' bronchiolitis differs from > 6 months' bronchiolitis. Consequently, we believe that is time to reach an agreement on the definition of bronchiolitis, as the first episode of acute lower airways infection, characterized by acute onset of respiratory distress with cough, tachypnea, retraction, and diffuse crackles on auscultation in infants <12 months, or even 6 months of age.

## What is Known

- Bronchiolitis is a common cause of lower respiratory tract infection in infants and the major responsible factor of hospitalization under the age of one year.
- Respiratory syncytial virus is major involved virus in bronchiolitis

## What This Paper Adds

- Infants younger than 6 months had more frequently respiratory syncytial virus infection and a more severe disease.
- Infants older than 6 months had slightly more tobacco smoking exposure and slightly higher recurrent wheezing after the hospitalization.
- The human bocavirus has been identified almost entirely in infants older than 6 months.

## Data Availability Statement

The datasets generated for this study are available on request to the corresponding author.

## Ethics Statement

The study was reviewed and approved by the institutional review board at Policlinico Umberto I (Ref: 108/2012). Written informed consent was obtained from the parents/legal guardians.

## Author Contributions

RN: analysis performance, interpretation of data for the work and drafting the work. AF, LP, and GD: acquisition of the data. FM: conception and design of the work.

## Conflict of Interest

The authors declare that the research was conducted in the absence of any commercial or financial relationships that could be construed as a potential conflict of interest.

## Abbreviations

hBoV: human bocavirus
hMpV: human metapneumovirus
hRV: human Rhinovirus
NPW: Nasopharyngeal washing
PCR: polymerase chain reaction
RSV: respiratory syncytial virus.
